# Microbial Degradation of Herbicide Residues in Australian Soil: An Overview of Mechanistic Insights and Recent Advancements

**DOI:** 10.3390/toxics13110949

**Published:** 2025-11-03

**Authors:** Imtiaz Faruk Chowdhury, Gregory S. Doran, Benjamin J. Stodart, Chengrong Chen, Hanwen Wu

**Affiliations:** 1 School of Agricultural, Environmental and Veterinary Sciences, Charles Sturt University, Wagga Wagga, NSW 2678, Australia; gdoran@csu.edu.au (G.S.D.); bstodart@csu.edu.au (B.J.S.); 2 Department of Agronomy, Sher-e-Bangla Agricultural University, Sher-e-Bangla Nagar, Dhaka 1207, Bangladesh; 3 Australian Rivers Institute, School of Environment and Science, Griffith University, Nathan, QLD 4111, Australia; c.chen@griffith.edu.au; 4 NSW Department of Primary Industries and Regional Development, Wagga Wagga Agricultural Institute, Wagga Wagga, NSW 2650, Australia; hanwen.wu@dpird.nsw.gov.au

**Keywords:** herbicide residue, persistence, microbial degradation, soil microorganisms

## Abstract

Herbicides are chemical compounds that are toxic to weed plants. Modern agriculture relies heavily on herbicides for the control of weeds to maximize crop yields. Herbicide usage in the Australian grains industry is estimated to have increased by more than 65% from 2014 to 2024, which equates to more than AUD 2.50 billion dollars per year. The increased popularity of herbicides in farming systems has raised concerns about their negative impacts on the environment, human health and agricultural sustainability due to the rapid evolution of herbicide resistance, as well as their behaviour and fate in the soil. Due to excessive use of herbicides, soil and water pollution, reduced biodiversity and depression in soil heterotrophic bacteria (including denitrifying bacteria) and fungi are becoming increasingly common. Biological degradation governed by microorganisms serves as a major natural remediation process for a variety of pollutants including herbicides. This review provides a brief overview of the present status of herbicide residues in Australian farming systems, with a focus on the microbial degradation of herbicides in soil. It highlights key bacterial and fungal strains involved and the environmental factors influencing the biodegradation process. Recent advancements, including the application of omics technologies, are outlined to provide a comprehensive understanding of the biodegradation process.

## 1. Introduction

Weeds are classified as an important biological constraint to food production and one of the major factors that cause yield reduction [1]. They compete with crops for available resources, such as nutrients, light, water and space, and threaten sustainable crop production. Therefore, weed control is critical with respect to increased crop production and requires investigation of both the active weed population and the soil seedbank [2]. Several techniques are commonly practiced to destroy or suppress weed populations for minimizing competition in crop fields, which attempt to maintain a balance between cost and yield loss. Considering the variety of options available, weed control is generally labour-intensive, including manual weeding and various mechanical control techniques. Chemical weed control is the most common approach due to low cost as well as less labour involvement than other approaches [3]. Chemicals that are used to control, suppress, or kill plants, or to severely interrupt their normal growth processes, are called herbicides [4], and this group of chemicals can efficiently control weeds with minimum energy requirements [5]. As an example, application of 2,4-dichlorophenoxyacetic acid (2,4-D) with a single knapsack sprayer was more effective and efficient than 15 labourers weeding with a hoe in sugarcane fields [6]. The use of atrazine made corn cultivation possible and profitable in some parts of the world by reducing costs and increasing the land area that farmers could grow and manage four-fold [7]. The rapid development and adoption of herbicides significantly contributed to global food production, but this has been offset by the increasing development of resistance by weeds, as well as the non-target toxicity of herbicides [8].

Herbicides can move vertically to contaminate groundwater via leaching or laterally to intrude into surface water. The presence of herbicides in surface and groundwater is of great importance considering the potential impact on human health and to the environment [9]. Residual herbicides may also be toxic to sensitive plant and animal species or enter the food chain due to accumulation in grains and crops [10].

Therefore, this article aims to provide a comprehensive understanding about the persistence of herbicide residues in soil. The main objectives are to summarize the present status of herbicide residues in Australian soil, outline the mechanisms involved in microbial degradation, review recent research progress, and discuss factors that influence microbial degradation of herbicide residues in soil. The study also highlights the potential of microbial degradation as an environmentally safe approach to reduce herbicide persistence and its negative effects.

## 2. Herbicides in Australian Agriculture

**Figure 1:**
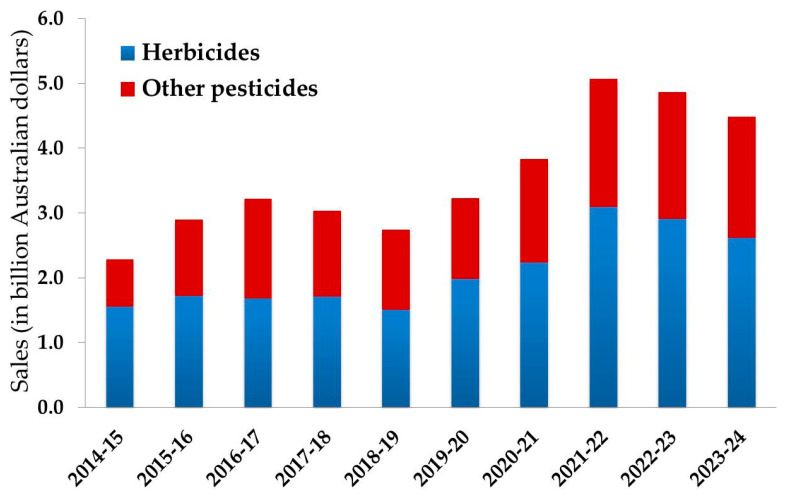
Year-wise breakdown of herbicide sales in Australian agriculture over total pesticide expenditure, expressed in billions of dollars [15].

Australian farming systems have evolved over the past 50 years with the adoption of conservation tillage, including minimal or zero till, which has reduced cultivation practices for weed control [11]. This evolution has led to the foundation of modern technologies in crop production, reducing cultivation practices and also providing fewer available options for weed control. Reduced cultivation practices favour weed growth which ultimately increases dependence on chemical weed control [12]. Other factors could also significantly contribute to the increased adoption of herbicides, such as the reduced price of the predominant herbicide, glyphosate, which was responsible for the rapid adoption of zero tillage among 78% of farmers in 2008 [13]. Research revealed that zero tillage combined with herbicide use in wheat reduced weed biomass by 45% and improved grain yield by 20% [14]. Statistics showed that herbicide usage in Australian farming systems is gradually increasing every year (Figure 1).

At present, Australian farmers spend more than 2.50 billion dollars per year on weed control, which is approximately 60% of the total annual national pesticide expenditure [15]. The increased popularity of herbicides in farming systems has raised concerns about their negative impacts on the environment, human health and agricultural sustainability due to the rapid evolution of herbicide resistance. In Australia, herbicide-resistant weed populations are prevalent compared to other major grain-producing countries [16]. The adaptation and evolution of herbicide resistance have enabled scientists and growers to radically rethink existing weed management approaches due to the lack of diversity in their weed control programmes. Increased use of pre-emergent herbicides could bring diversity in the weed control programme. It is estimated that the use of pre-emergent selective herbicides in Australian winter broad acre crops has risen from less than 1 million ha to approximately 7 million ha in a decade [17]. The increased use of herbicides in agricultural fields leads to the accumulation of herbicide residues in soil, which requires appropriate management strategies. Development of efficient and sustainable remediation techniques is essential for safe crop production and to reduce the risk of environmental contamination.

## 3. Significance of Herbicide Residues in Australian Soil

Herbicides are applied to soil to control unwanted vegetation that can interfere with the growth and development of commercial crops. The period of time an herbicide remains active in the soil is considered its persistence and is ideally expected until the end of the cropping period. However, existing soil conditions, herbicide chemical structure, as well as the application method, will determine the persistence of herbicides in soil [18]. Long-term persistence of herbicides may lead to soil and ground water contamination [8], affect biodiversity and decrease soil heterotrophic bacteria (including denitrifying bacteria) and fungi [19]. Herbicide residues applied in minimal or zero till systems tend to remain more concentrated near the soil surface at the end of the cropping season [20]. Some herbicides can remain in soil for weeks, months or even years, which may be considered advantageous in regard to long-term weed control. However, occurrence of herbicide residues in higher concentrations may affect subsequent crops [10] due to their residual activity in subsequent years, which can limit planting options for farmers.

Regular monitoring of pesticide residues in Australian soils has been undertaken over decades, leading to the Australian government to ban organochlorine herbicides in late 1970s due to their long-term persistence in soil. More recently developed herbicides tend to show lower persistence in soil. To better understand and manage herbicide residues in the soil, surveys from different crop fields from around Australia detected residues of 23 chemicals, with glyphosate and its primary metabolite, aminomethylphosphonic acid (AMPA), being most frequently detected, followed by trifluralin, 2,4-D, diflufenican, atrazine, etc. [21].

The increased use of glyphosate worldwide over the last few decades led to more research focussing on the potential persistence of glyphosate in soil [22]. Following the world trend, glyphosate (15%) was the most frequently applied herbicide, followed by trifluralin (10%), 2-Methyl-4-chlorophenoxyacetic acid (MCPA) (9%), paraquat (7%) and triasulfuron (7%) in Western Australian cropping systems from 2010 to 2014 [23]. According to [21], glyphosate and AMPA were frequently detected over two years (67 and 93% of samples, respectively), with median concentrations of 0.22 mg kg/ha and 0.31 mg kg/ha, respectively, in 2016. Maximum concentrations of glyphosate detected in topsoils worldwide are comparatively higher than those found in Australian soils. For example, <1.50 mg kg/ha was reported in Argentina soil [24] compared to 2.00 mg/kg in European soil [25]. Contributing to this variety of results from various geographic locations, several analytical procedures have been used to reflect fast and reliable quantification of the contaminant, with variable limits of detection, quantification and accuracy that may influence the results [26]. Another possible explanation is the majority of glyphosate applied as a pre-sowing application and summer fallow spray in Australian farming systems [23] compared to the greater use of glyphosate-resistant crops in other parts of the world [26]. Moreover, prolonged drought conditions, along with strong adsorption, would facilitate persistence of glyphosate and AMPA in Australian agricultural soils due to reduced microbial activity [27].

Trifluralin was also frequently detected (>50% sampling frequency) both in 2015 and 2016 across Australia, but the maximum residue concentration (5.35 mg/kg) in 2016 was considerably higher than 0.59 mg/kg in 2015 [21]. Trifluralin is widely used in Australian farming systems to control grassy weeds. It is well-known for long persistence in soil, having half-lives of 35 to 375 days under field conditions [28] compared to 5.80 to 26.74 days under laboratory conditions [29]. Moreover, trifluralin has been reported to have a carryover potential of 9–24% from one season to next in Australian farming conditions [30], which increases up to 90% carryover under prolonged drought conditions [31]. This agrees with [29], who concluded that trifluralin dissipation in soil may be addressed as a function of soil temperature and moisture.

Diflufenican has also been detected frequently but at lower concentrations in a two-year survey of Australian crop fields [21]. It is also a frequently used as a pre-emergence herbicide, with a long half-life of 224 to 621 days in soil [28], similar to trifluralin. The higher detection rate of diflufenican reflects its strong retention in soil reported by [32] and frequency of use in Australian farming systems. According to [33], diflufenican was one of the most frequently detected pesticides in France, with a median concentration of 0.14 mg kg/ha in crop soil. However, no carryover issues were observed with continuous diflufenican application for four years in central and northern Italy, possibly due to rapid microbial degradation [34]. The herbicide 2,4-D was frequently detected (94% sampling frequency) in 2016 in Australian soils, but at lower concentrations, mainly due to its common use in summer fallow and the winter season [21]. 2,4-D is considered to be non-persistent in nature when microbial activity is sufficient [35]. In addition, atrazine residues were also detected in higher concentrations of New South Wales and South Australian field soils compared to other regions [36].

Pesticides are commonly applied in combinations, with approximately 70% of crop fields worldwide (i.e., about 8.31 million km^2^) containing multiple pesticide residues in the topsoil [37]. Ref. [21] found that herbicide residue mixtures were prevalent in arable Australian cropping soils, with an average of 6–7 different herbicide residues per soil sample, resulting from an average of 6.3 herbicide applications per year to each field in Western Australian cropping systems [23]. Similarly, Ref. [38] observed a median of nine herbicide residues in arable soils under no-till or conventional management in Switzerland, which is likely due to most European cropping fields being reported as having higher numbers of different pesticide residues than in Africa, South and Southeast Asia, and Australia [37]. Moreover, the agro-climatic conditions of Europe, USA and Latin America are not comparable with the Australian context. Herbicide persistence in soil is directly related to the dissipation behaviour of the specific herbicidal compound where chemical, environmental and soil properties play important roles in determining their fate [29]. Therefore, understanding the fate of herbicides in the soil is a prerequisite for the accurate assessment of their behaviour and potential environmental risk [39].

## 4. Distribution and Functions of Microorganisms in Soil

Microorganism is a broad term that includes bacteria, fungi, archaea, protists and viruses. While typically representing about 0.1% of the total volume of soil, microorganisms are largely responsible for processes that recycle organic waste and pollutants present in the soil [40]. They are present in soil regardless of the textural classes and types, but their population densities will differ. For example, one gram of soil may contain more than 10^9^ bacterial and archaeal cells and 200 m of fungal hyphae in the surface layer [41]. Microorganisms have been reported to play a vital role in waste decomposition [42], regulation of plant growth [43], nutrient cycling [44] and the degradation of various dangerous contaminants and pesticides [45]. Microbial degradation/biodegradation is a process through which toxic/pollutant/chemical compounds are rendered benign or less toxic than their parent compounds by microorganisms like fungi and bacteria [46]. Recent studies suggested the need for rapid exploration of novel microorganisms, their diversity and innovative ecological functions for the development of bioremediation strategies [47,48,49,50].

Microbial distribution in soil is regulated by several biotic and abiotic factors. Modifications to environmental conditions may alter the equilibrium distribution of microbial populations, which may be responsible for the differences in adaptability of microbial populations in different geographical locations [51]. For instance, the abundance, composition, diversity and enzymatic activity of microorganisms present in the rhizosphere can be expressed as a subset of overall soil microbial community, which is influenced by the localized physiochemical properties of the soil [52], which may be different from the bulk soil [53]. This is reflected where plant root exudates have been reported to shape the composition and abundance of the rhizosphere microbial community [54].

## 5. Microorganisms Involved in Microbial Degradation

The over-reliance of pesticides in agriculture may lead to the accumulation of toxic compounds in the environment, driving the need for soil remediation. Microorganisms are responsible for the enzymatic transformation of toxic compounds into non-toxic compounds in soil [55]. They possess biological and catalytic activity that may result in the degradation of soil pollutants via the extra-cellular secretion of enzymes that are responsible for their degradation [56]. Some bacterial enzymes are able to decompose aromatic and cyclic chemicals into simple compounds for transport across the cellular membrane for metabolism [57]. The differences in degradation are due to the sensitivity of bacterial species to the chemical compounds. Bacteria predominate the microbial community regardless of the soil depth, as they can utilize alternative electron acceptors in oxygen-deficient conditions [58]. They have the greatest capability to produce new metabolic pathways due to the evolution of new enzymes that permit the degradation of resistant chemicals [59]. Bacteria are able to transfer clusters of genes evolved in a bacterium to other organisms by cell-to-cell contact, which is known as horizontal gene transfer [60]. This approach allows the development of a protection system against toxic pollutants due to the continuous exposure to various environmental stresses, allowing bacteria to take advantage of a broader variety of carbon compounds [51,61]. This property is more common in bacteria but can also occur in other organisms, where bacteria serve as a donor of genetic material, while fungi, plants and animals may serve as recipients [62]. There is significant evidence of the generation of new bioremediation pathways within the microbial community by the transmission of genes responsible for biodegradation [63]. Ref. [64] reported the intra-field evolution and inter-field exchange of 2,4-D catabolic plasmids and genes within a restrained local environment. Various soil bacterial genera have been identified that are involved in the degradation of hazardous chemicals in soil [65]. Table 1 represents the list of bacterial and fungal strains associated with the degradation of various herbicides, rate of degradation and place of isolation in the last five (5) years (2019 to 2025).

Among all bacterial genera, *Bacillus* is known to have the greatest ability to efficiently degrade persistent pesticides [108]. *Bacillus atrophaeus* YQJ-6 was reported to tolerate extreme concentrations of atrazine (1000 mg/L) and can degrade approximately 99.2% atrazine in 7 days [109]. *Bacillus* genus has been reported to be highly adaptive in nature and capable to degrade a wide range of pesticides, with the evolution of mutants potentially leading to new metabolic pathways [110]. Ref. [111] identified a 2,4-D degrading gene cluster, *tfdII*, located on plasmid pJP4 of *Ralstonia eutropha*. Various species within *Pseudomonas*, *Arthrobacter*, *Alcaligenes*, *Cytophaga*, *Actinobacter*, *Moraxella* and *Klebsiella* have been reported to have such types of plasmids [112].

Fungi-mediated bioremediation has been reported to be satisfactory due to their extended mycelial networks, low specificity to catabolic enzymes and independency towards utilizing organic compounds as growth substrates [113]. Fungal degradation of herbicides is also regulated by a number of environmental factors including soil moisture [114], temperature [115,116], pH [116], aeration [117] and composition of the medium [118]. However, research suggests that fungi-mediated degradation is a slow process and generally does not result in the complete removal of the pollutants [119], possibly due to the time required for the adaptation of the fungal strain in a contaminated environment. Metabolic processes and mechanisms governed by biodegradation processes need to be addressed under variable environmental conditions which ultimately contribute to fungal biodegradation in site-specific conditions. The changes in environmental conditions will affect the physiology of the fungal species, ultimately affecting the degradability of the pesticides. Another important aspect regarding fungal biodegradation is that incomplete degradation of the pollutants may lead to the possibility of increased metabolite toxicity compared to the parent compound [120]. Accidental accumulation of those metabolites in soil may have serious consequences [121].

## 6. Mechanisms Involved in Microbial Degradation

Soil microbes are an indispensable part of the ecosystem, maintaining biogeochemical cycles through novel transformations in the biosphere [122]. As a part of the transformation process, various organic and inorganic compounds deposited in soil are converted to simple compounds through a variety of metabolic pathways adopted by specific microorganisms or groups of microorganisms. Under aerobic conditions, herbicides are primarily converted to CO_2_ due to oxidation, but other chemicals may also form. Microorganisms require energy for the various metabolic activities they perform in soil, and they mainly rely on the organic compounds as a source of energy. Since microbes catabolize herbicide compounds for assimilation as an energy source, their interaction with the herbicidal compounds is significant (Table 2). Catabolic metabolism is mainly dependant on the suitable chemical structure of herbicidal compounds to be utilized as an energy source by the microorganisms.

Co-metabolism is a process where herbicides are degraded by mechanisms used to degrade other organic chemicals, and the degradation rate will depend on both the concentration of herbicide and carbon source but typically occurs where the amount of herbicide is comparatively lower than the other available carbon sources. Ref. [127] reported an approach by increasing the concentration of the primary substrate for the degradation of stable and non-degrading chemicals. The consequences of adaptation and co-metabolism ultimately result in microbial degradation of the compound, which is of primary interest in understanding the mechanisms behind microbial activities in soil.

### 6.1. Adaptation

Microbial degradation of herbicides depends on the frequency of herbicide application to the soil. Repeated application of the same herbicide in the same field results in increased microbial degradation, suggesting adaptation as a result of selection [123]. As a result of accelerated degradation of herbicides, soil-applied herbicides are losing their efficacy [128], and microorganisms are hereby accounted for undermining the effectivity of herbicide compounds. Moreover, the ongoing debate regarding the accelerated degradation of glyphosate in soils with a decade-long application history has yet to be scientifically proven [129]. However, this rapid breakdown of herbicides has been reported to be disadvantageous by several researchers whereas others have described it as an uncommon phenomenon having little impact on agriculture [130].

**Figure 2:**
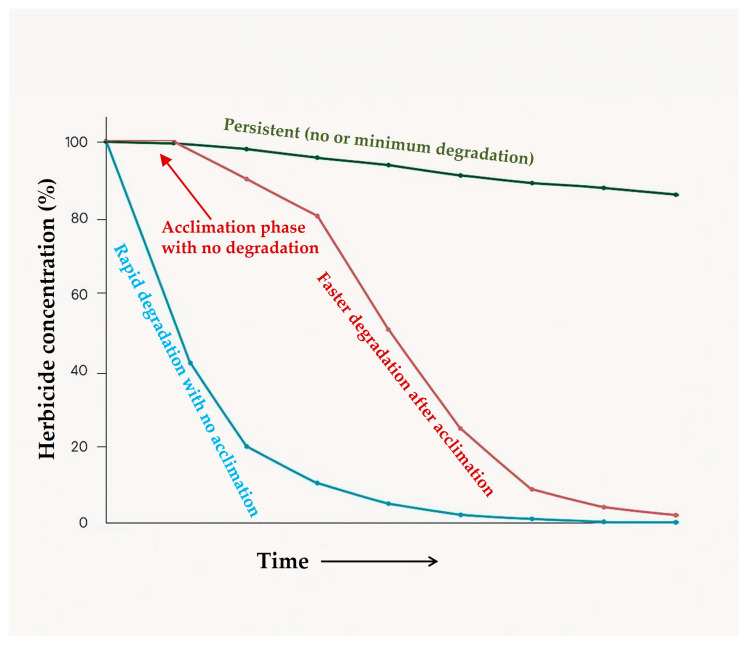
Degradation of herbicides in soil over a period of time. Updated from [138].

There is a range of opinion available to describe how microorganisms build up their capacity to degrade a certain herbicide. According to [131], a specific signal derived from the applied herbicide or other chemicals is responsible for the microbial adaptation to specific herbicides. Some chemicals may act as a motivator in enzyme secretion, which further degrades other chemicals. The degradation gene cluster *tfd* (*tfdABCDEF*) is involved in the gradual catabolic metabolism of 2,4-D and was initially identified in the plasmid pJP4 of *Cupriavidus necator* JMP134 [132,133]. Again, it is not mandatory for the herbicide compounds to be substrates for the metabolism process governed by enzyme secretion. Traditional microbial culture-based laboratory investigations mainly concentrated on the monoculture of substrates, but the complete degradation of herbicides is faster and more efficient in microbial consortia rather than a single microorganism [134]. Recent studies not only revealed the involvement of microbial communities in the remediation of toxicants in the soil but also demonstrated the interaction between different microbial species [40]. These innovative studies further laid the foundation of exploring the adaptation mechanism behind herbicide selectivity of the microorganisms in adverse conditions. Herbicide degradation usually shows an initial lag phase where no degradation occurs, followed by a sharp decrease in the concentration (Figure 2). The period between herbicide application and initiation of biodegradation is termed as the acclimation period where basically no significant degradation is observed. Ref. [135] observed a prolonged lag phase followed by higher concentrations of applied atrazine; however, repeated application of atrazine resulted in faster degradation with no lag phase [123]. This may be due to the multiplication of herbicide-degrading organisms during the first application to a level that increased the degradation rate of herbicides during later applications. Other proposals identified genetic alterations taking place within the microorganism for enzyme synthesis as the main reason for the initial time lapse [40]. Alterations are mainly due to changes in chromosomal or extra-chromosomal DNA sequences. A specific type of extra-chromosomal DNA, commonly known as plasmid, has been identified to be responsible for the degradation of herbicides [111]. These special types of DNA are smaller than bacterial chromosomes and have been reported to bear specific genes for biodegradation of herbicides which may be absent in chromosomal genes [133]. Plasmids are capable of intercellular movement in some microbial communities and provide a pathway for the transfer of the biodegrading genes to other members of the bacterial community [136]. For example, whole-genome analysis of 26 bacterial strains revealed horizontal gene transfer (HGT) events, suggesting that these strains may be integrating plasmids in their genomes through HGT to adapt to changing environmental conditions and are most likely involved in degrading toxic substances [137]. Manipulation and transportation of these genes from one organism to other members of the microbial community in such conditions has opened a new horizon in the context of bioremediation.

### 6.2. Co-Metabolism

Co-metabolism is the coincidental degradation of an herbicide by an enzyme or co-factor associated with the degradation of another compound [65]. The energy derived at this process is neither sufficient to support microbial growth nor to activate relevant enzymes involved in the degradation process [139]. Co-metabolism is mainly dependant on the substrate metabolism of other compounds. This type of biodegradation is highly sophisticated, as only the microorganisms capable to degrade the concerned contaminant are accelerated [140]. This process can be accelerated at low concentrations, particularly to an undetectable limit, i.e., parts per trillion, which is the most important advantage. For example, methanotrophs (prokaryotes that metabolize methane for their sole carbon and energy source) have been reported to produce an enzyme called methane monooxygenase, which is capable of oxidizing over 300 compounds [140]. These reactions often produce intermediate compounds, i.e., metabolites, that may serve as substrates for subsequent microbial taxa, minimizing persistence and toxicity [65]. In contrast, some evidence suggests that metabolites produced from this specific type of metabolic pathway may act as inhibitors of microbial degradation [141]. Microbial co-metabolism may be the effective approach to remove various types of toxic pollutants from soil [139]. According to [40], majority of the herbicides used in agriculture may be degraded by co-metabolism.

Research suggests that no lag phase has been observed in co-metabolic pathways [142]. Moreover, adaptation is absent in co-metabolism, which indicates that repeated application of herbicides does not affect co-metabolic degradation at all. Synthetic chemicals which are not degraded by individual microbial species may be mineralized further via the co-metabolic transformations governed by the combined activity of several microbial species. Co-metabolism of herbicides generally occurs at a slow rate due to the lower populations of co-metabolizing microorganisms, which will not increase with respect to the chemicals applied [143]. Even so, a single microorganism can co-metabolize a pollutant completely [144]. These co-metabolizing microorganisms can be considered as a good option for the development of bioremediation strategies.

## 7. Factors Affecting Microbial Degradation

### 7.1. Temperature

Temperature plays a major role in the ecological distribution of microorganisms associated with metabolic activities and the degradation of herbicides in soil [145]. Ref. [146] demonstrated the rapid increase in soil microbial respiration with increasing temperature, which could accelerate degradation of pollutants such as herbicides [147]. Laboratory incubations for herbicide degradation are often conducted at 25 °C and may not reflect genuine soil temperatures; therefore, they may not be reflective of genuine microbial activity [148]. Ref. [149] showed that the optimum temperature range for atrazine degradation was 20–40 °C, and lower temperatures tend to result in the accumulation of toxic pollutants in the environment [150]. Ref. [151] reported that mesosulfuron-methyl may persist in soils at low temperatures. In contrast, Ref. [152] attributed the accumulation of isoproturon in soil under dry and hot weather conditions to drastic changes in soil microbial community structure and function. Rapid degradation of clopyralid was observed by [153], possibly due to higher temperatures (14.4 and 16.9 °C) in Alaskan soils. Temperature has been shown to regulate the enzymatic activities required for various biochemical processes in soil [154]. According to [155], enzyme activation mainly depends on the physical and chemical interactions with soil clay, minerals and organic matter. Studies related to the effect of temperature on the degradation of herbicide compounds found that higher or lower than the optimum temperature will slow down the degradation process [29]. The optimum temperature for herbicide degradation may vary between chemicals but will generally be in the range of 20–30 °C [156]. Ref. [157] reported that atrazine residue concentration decreased with increasing temperature as a result of increasing the degradation rate, and half-life was decreased by 3–4 times from shifting the temperature from 5 °C to 35 °C. Degradation of trifluralin and atrazine was strongly influenced by temperature, with half-lives ranging from 5.8 and 7.5 days at 30 °C to 22.44 and 25.84 days, respectively, at 10 °C [29].

### 7.2. Soil Moisture

Soil microorganisms require moisture for their growth and metabolism. There is a direct relationship between soil microbial activity and moisture content; a decrease in moisture content reduces microbial activity, and rewetting causes a large and rapid increase in activity [158]. Therefore, the degradation process is slow in dry soils and generally increases with increasing moisture content [159]. This may be due to the low microbial activity prevailing under extreme dry conditions [160]. Since moisture content has a significant impact on soil microbial activities, herbicide degradation would be expected to be faster in wet soils. Generally, moisture contents between 50 and 80% field capacity (FC) levels are considered optimal for microbial activity [29]. Atrazine degradation was reported to be 3–4 times higher when soil moisture content was increased from 5% to 20% [157].

Whereas extreme soil moisture conditions are considered unfavourable for microbial growth and metabolism process, fungal and bacterial oxidative enzymes for degradation are inhibited at low O_2_ levels in saturated soils. Excess moisture can accelerate anaerobic transformation of herbicides by reducing the oxygen level, which could hamper the transformation of herbicides [161]. In contrast, the complete absence of oxygen is required for the initial dechlorination of chlorophenoxyacetic acids by *Dehalococcoides mccartyi* [101]. However, soil moisture may not necessarily have any significant effect on the transformation of some herbicides, e.g., atrazine [29]. Some herbicides are reported to be accumulated under anaerobic conditions, e.g., clopyralid [162], whereas others breakdown rapidly, e.g., S-metolachlor [159].

### 7.3. Soil pH

Soil pH can have substantial effects on the reactivity, activity and persistence of applied herbicides in soil, specifically at extreme pH conditions such as less than 4.5 or higher than 7.5 [163]. The basic principle is that herbicide degradation is dependent on the charge of the herbicide molecules. Herbicides bearing a positive charge will have a strong affinity to negatively charged soil clay particles, caused increased persistence in soil [164], whereas herbicides bearing a negative charge will be repelled by soil colloids and exposed to transformation [165]. Ref. [164] demonstrated that imazethapyr dissipated faster under alkaline soil followed by neutral and acidic soil, indicating that imazethapyr has a longer residence time in acidic soil. Similarly, stronger adsorption of herbicide pelargonic acid has been observed under low pH conditions [166]. Optimum conditions for the biodegradation of herbicides will vary with herbicide and the soil microorganisms, but the degradation rate is slow at acidic pH compared to alkaline and neutral pH conditions because acidic pH increases the stability of various chemical groups [167]. Another possible reason may be the reduced activity of bacteria or enzymes involved in the biodegradation process under low pH [168].

Again, soil pH has a major influence not only on the growth and activity of microorganisms but specifically on the growth of microbes responsible for herbicide transformation [169]. Optimization of pH in soil is a difficult task depending on the soil type, as variation in the soil pH is comparatively less than in water. In addition, enzymes have an operational pH range, and changes to pH cause inhibition due to denaturation. As most microbial species survive in the pH range of 4.5–7.5 [170], optimization of pH is critical in soil experiments in regard to biodegradation of herbicides [171]. Ref. [126] observed the highest degradation of atrazine in soil when pH was increased from 5 to 7; further increasing pH from 7 to 9 reduced degradation, which may be due to loss of bacterial activity at slightly acidic and slightly basic conditions. Accelerated biodegradation of endosulfan was reported through optimization of pH to 8.0 [172].

### 7.4. Soil Organic Matter

Although microorganisms represent only a small portion (<5%) of the soil organic carbon [173], they are responsible for the maintenance of C, N and P cycles and other physio-chemical activities in soil through decomposition, mineralisation and immobilization processes [174]. Soil organic matter acts as the main factor in herbicide sorption for non-ionic herbicides and therefore regulates their concentration in the soil solution as well as their transport down the soil profile [175]. Trifluralin strongly adsorbs the soil organic matter due to its hydrophobic nature, which decreases its bioavailability to soil microbes for degradation [176]. To boost microbial activity in soil, organic matter content in soil should be replenished [177]. According to [178], soil organic matter concentrations should be at least 1.0% to ensure the activity of indigenous microorganisms involved in the transformation of toxic compounds in soil. To increase the organic matter content in soil, application of various organic amendments such as sawdust, municipal waste compost and synthetic biological waste is frequently practiced in different countries [179,180,181,182]. The addition of organic amendments will either slow down the microbial degradation process through adsorption [183] or accelerate the remediation process by increasing the microbial metabolic activity [184]. Organic amendments, e.g., sawdust, have higher C:N ratios than compost, resulting in increased microbial activity because microorganisms require carbon and nitrogen as nutrients for growth and reproduction [185]. The addition of organic amendments in the soil has recently gained increasing interest [186]. However, this could lead to a change in the fate and behaviour of herbicides applied in the same soil [187]. One possible option to reduce the leaching of herbicides could be achieved through improving herbicide sorption by increasing the soil organic matter content, which will improve soil quality [188]. Ref. [189] reported that soil amendment with green compost not only increased the half-life (DT_50_) of triasulfuron in soil due to rapid adsorption by soil particles but also accelerated persistence by blocking leaching into the soil.

### 7.5. Herbicide Structural Properties and Concentration

Physical and chemical properties of herbicides will dictate the rate of their biodegradation in the environment. Simple, low-molecular-weight compounds have been reported to degrade faster than larger polymer and composite compounds [190]. The addition of polar groups such as, OH, COOH and NH_2_ on the phenyl ring makes the herbicide more susceptible to microbial activity [191]. On the other hand, Ref. [192] revealed that substituents like halogen and alkyl groups make compounds resistant to microbial degradation. In addition, water solubility and adsorptivity of herbicides are inversely related for many herbicides. Herbicides with high water solubility are more prompt to microbial degradation than those which have low water solubility [193]. Dinitroaniline herbicides have poor solubility and can easily adsorbed by the reactive groups of soil organic matter in the soil, making them difficult to measure and not able to readily degrade [191]; however, glyphosate and paraquat exhibit high water solubility but are adsorbed tightly to soil particles [194]. Several physical, chemical and structural parameters that determine the possibility of herbicide degradation are listed in Table 3.

### 7.6. Dissolved Organic Matter (DOM)

Dissolved organic matter (DOM) is the fundamental portion of organic matter with the ability to dissolve in field conditions and which plays a major role in the transportation of pollutants in soil [195]. Photosynthesis is the primary driver of DOM production in soil, which includes the degradation of organic litter and humus substances accumulated through pedogenesis [196,197]. Soil microbial communities are the substantial agent contributing to the formation of DOM. Ref. [198] investigated DOM structure and fractionation and revealed that microorganisms transform plant-derived compounds, leaving DOM to become increasingly dominated and difficult to degrade compounds as degradation proceeds. Solubility and mobility of various organic compounds and herbicides are enhanced by DOM [187], followed by accelerated biodegradation such as, 2,4-D [199]. Microorganisms may use molecules present in natural DOM directly or indirectly, accelerating biodegradation [200]. According to [201], DOM serves as both a source of carbon and provides energy for microbes involved in biodegradation processes because microbial biomass, community structure and microbial functions are closely related to the property and molecular composition of DOM. The interaction of DOM with herbicides affects herbicide bioavailability, mobility and ultimately the effectiveness of microbial degradation. Moreover, in return, microbial metabolic activity affects the transformation of specific types of compounds in DOM (e.g., proteins), thus also contributing to the total DOM pool, resulting in dynamic changes in DOM properties and composition [201]. Studies have shown that DOM stimulates the degradation of herbicides (linuron) by providing supplementary carbon that supports potential degraders [202]. However, in another study, Ref. [203] reported that DOM supply was not stimulating glyphosate degradation by microbial heterotrophs possibly due to the low production of dissolved organic carbon and complex interactions between microbial groups that limit glyphosate breakdown despite the presence of DOM.

Most studies focussed on the behavioural pattern of herbicides in water bodies while very little is known about their interaction in soil in the presence of DOM [204]. Adsorption and desorption behaviour of herbicide atrazine was also influenced by the nature of DOM as per [205]. They reported that application of DOM reduced atrazine sorption and significantly increased atrazine desorption from the soil. The reduced sorption may be attributed to the complex formation of DOM with organic chemicals or competition for sorption sites.

## 8. Approaches Used to Study Microbial Degradation

**Figure 3:**
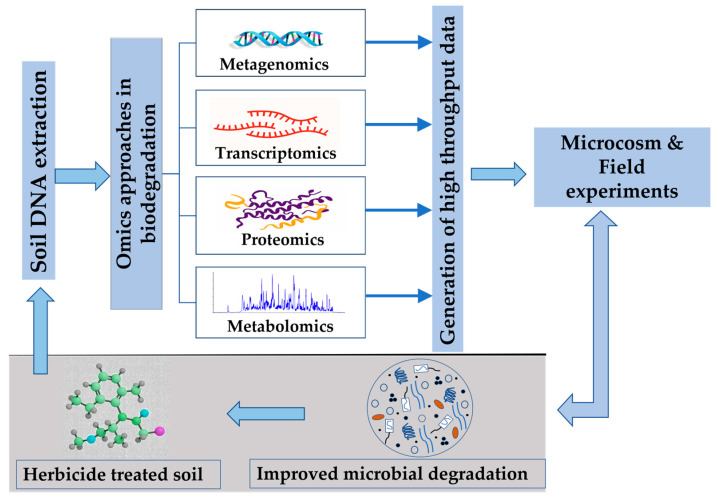
Salient features of omics-based technologies for the degradation of persistent herbicides in soil.

Recent developments in microbiology have allowed us to use various molecular “omics”-based approaches such as genomics, proteomics, transcriptomics, metabolomics, fluxomics, etc., to investigate specific microbial catabolic pathways for the biodegradation of herbicides [206]. Using advanced omics technologies, researchers can analyze changes in the expression of proteins involved in pesticide degradation (proteomics) to detect and quantify metabolites formed during the degradation process (metabolomics) and to examine alterations in gene expression triggered by microbial exposure to pollutants (transcriptomics) [207]. Genomic and metagenomic approaches focus on identification of novel microorganisms responsible for the degradation of pollutants through providing insights about the genetic makeup and metabolic capabilities of the microbial communities involved in bioremediation [208]. The generated high-throughput data from omics-based technologies could potentially be applied in laboratory and field experiments to improve biodegradation studies of persistent herbicides in soil (Figure 3).

The absence of degrading microorganisms could make the scenario difficult, favouring the herbicide compound persisting in soil longer than usual. Transcriptomics and proteomics reveal molecular mechanisms responsible for pollutant degradation through identifying the genes and proteins that are actively expressed during microbial degradation [209]. The latest omics-based technologies used to study microbial degradation of herbicides are listed in Table 4.

Catabolic genes play an important role in shaping the genetic foundation of herbicide biodegradation, with subsequent identification of these genes permitting application of molecular technology to investigate their function [221]. Catabolic genes are generally situated on chromosomes, but several have been located on plasmids. More than 300 genes have been isolated to date from various culturable bacterial strains responsible for biodegradation of aromatic compounds [222]. Greater emphasis has been given to DNA and RNA quantification to identify the number of potential biodegrading genes. It is believed that a positive correlation may exist between the relative abundance of biodegrading genes and their ability to degrade contaminants in the environment. Quantitative studies related to DNA and RNA can significantly promote biodegradation of herbicides by identifying the bulk of genes associated in bioremediation. This can allow the manipulation of the environment to promote the increase in the number of organisms involved in biodegradation. Ref. [223] provided new insights into the process of sulfonylurea herbicide degradation through identification of the gene CarE in *Rhodococcus erythropolis* D310-1 involved in the catalysis of chlorimuronethyl de-esterification through the catalytic action of carboxylesterase. Genomic approaches in bioremediation research have added new insights by regulating the rate and extent of contaminant breakdown more effectively. In a recent study, herbicide mesotrione was reported to be degraded by a novel *Klebsiella pasteurii* strain with exceptional adaptability to environmental conditions. Genome sequencing further revealed that nitroreductase-encoding genes *nfsA* and *nfsB* are mainly responsible in mesotrione biodegradation [224]. In another study, the discovery of a new amidohydrolase gene, AmiH52, expressed in *E. coli*, demonstrated both esterase and amidohydrolase activities to degrade various amide herbicides, e.g., propanil [225].

Researchers are placing more emphasis on the sequencing of whole genomes from a wide range of microbial populations in the soil to investigate novel genes and degradative elements responsible for the degradation of herbicides. Whole-genome sequencing enables detailed identification of microbial taxonomy and pathways, from strains to broader taxonomic groups involved in microbial degradation of pesticides under both aerobic and anaerobic conditions [226]. This has provided new insights into the identification of herbicide-degrading genes from both culturable and non-culturable microorganisms and provided an increased understanding of innovative metabolic pathways under various environmental conditions, which is essential for the successful implementation of bioremediation techniques [227].

Recent research approaches pointed out the potential of engineered bacteria for herbicide degradation [228]. These genetically modified super microorganisms may degrade the herbicide faster than the usual. However, the engineered bacterium was reported to degrade 100 mg/L fomesafen with a degradation efficiency of 82.65 % on the 7th day in the inorganic salt medium, which was 3.53 % lower than the degradation rate of the original strain of *Fusarium verticillioides* [220]. This warrants continued optimization and rapid exploration of genetically engineered bacteria for future advancements. Moreover, the adaptability of the microorganisms in the contaminated site may be an issue which could lead this strategy to be ill-fated. Additionally, the potential risk associated with genetically modified microorganisms in open environments raises common safety concerns and legislative issues [228].

## 9. Conclusions

In Australia, herbicides applied in minimal or zero till systems tend to maintain a greater concentration of herbicide near the surface zone at the end of the cropping season, which may result in higher residual concentrations, affecting crops subsequently sown. Currently, there is no option available to combat this problem other than using crop rotation to avoid incompatible crop–herbicide combinations, which includes routine rotation of fallow and pre-emergent herbicides, reliable record keeping helping to identify potential residue issues and the use of tolerant crops or crop cultivars in rotations after dry seasons. Due to a lack of proper monitoring, information about herbicide residues in Australian soil is limited. The persistence of herbicides in soil could potentially affect sensitive crops in rotation; thus investigations on critical concentrations at which levels cause damage to sensitive crops could help farmers in selecting crop rotation strategies. In addition, environmental factors are known to have a crucial effect on the persistence of herbicides in soil. Again, the persistence of herbicides in soil is directly related to the presence or absence of degrading microorganisms in soil; the shift in microbial community structure and diversity upon herbicide exposure could generate valuable information about potential groups of microorganisms benefitted by herbicide application.

Despite the widespread use of herbicides and, consequently, their undesirable presence in the environment, microbial degradation pathways of herbicides and their genetic bases remain poorly understood. Enzymes form a critical aspect in the degradation process as the degradation of the herbicide compound is governed through this. Since enzymatic degradation of herbicides poses as a promising approach, the necessity of extensive research regarding identification of degradable enzymes should be given utmost priority. Microbial communities possess greater genetic and metabolic diversity compared to a single strain in the degradation process. Moreover, the genetic expression and efficiency of the metabolic pathways are largely determined by the native environmental factors. So, priority investigations should be carried out for the identification and isolation of target genes considering native environmental factors so that the generated data could be applied in actual field conditions for the successful removal of herbicide residues in soil.

## Figures and Tables

**Table 1:** List of isolated bacterial and fungal strains associated with herbicide degradation in the last five years (2019 to 2025).

Isolated Strains	Herbicide(s) Degraded	Degradation Rate (%)	Location/Source of Isolation	Reference(s)
**Bacteria**				
*Arthrobacter* sp. SVMIICT25, *Sphingomonas* sp. SVMIICT11 and *Stenotrophomonas* sp. SVMIICT13.	2,4-D	81–90% after 12 days	India	[66]
*Cupriavidus oxalaticus* strain X32	2,4-D	100% in 3 days	China	[67]
*Bacillus cereus*	MCPA	99.70% on 31 days	Poland	[68]
*Pseudomonas fluorescens*	Sulfosulfuron	97% on 46 days	Poland	[68]
*Bacillus subtilis*, *Rhizobium leguminosarum*, *Streptomyces* sp.	Glyphosate	85–90% in 30 days	India	[69]
*Nocardia mediterranie* THS 1	Glyphosate	ND	India	[70]
*Amycolatopsis* sp. and *Saccharomonospora* sp.	Acetochlor, S-metolachlor	ND	China	[71]
*Bacillus aryabhattai* FACU3	Glyphosate	ND	Egypt	[72]
*Achromobacter denitrificans* SOS5	Glyphosate	56% after 96 h	Argentina	[73]
*Achromobacter insolitus* SOR2	Glyphosate	47% after 96 h	Argentina	[73]
*Achromobacter xylosoxidans* SOS3	Glyphosate	37% after 96 h	Argentina	[73]
*Agrobacterium tumefaciens* CHLDO	Glyphosate	40% after 96 h	Argentina	[73]
*Ochrobactrum haematophilum* SR	Glyphosate	41% after 96 h	Argentina	[73]
*Bacillus megaterium*	Glyphosate	70–71% after 60 days	Iraq	[74]
*Acidovorax* sp. CNI26	Glyphosate	100% after 125–400 h	France	[75]
*Agrobacterium tumefaciens* CNI28	Glyphosate	100% after 125–400 h	France	[75]
*Ensifer* sp. CNI115	Glyphosate	100% after 125–400 h	France	[75]
*Novosphingobium* sp. CNI35	Glyphosate	100% after 125–400 h	France	[75]
*Ochrobactrum pituitosum* CNI52	Glyphosate	100% after 125–400 h	France	[75]
*Comamonas odontotermitis* P2	Glyphosate	90% after 104 h	Australia	[76]
*Ochrobactrum* sp.	Glyphosate	60% after 15 days	Mexico	[77]
*Pseudomonas citronelloli*	Glyphosate	60% after 15 days	Mexico	[77]
*Lysinibacillus sphaericus*	Glyphosate	79% after 30 days	Colombia	[78]
*Aspergillus oryzae* AM1	Glyphosate	57% after 15 days	Argentina	[79]
*Stenotrophomonas* sp., *Brucella* sp., *Ensifer adhaerens*	Atrazine and metribuzin	ND	Iran	[80]
*Escherichia fergusonii*	Diuron	ND	Brazil	[81]
*Bacillus altitudinis* A16	Butachlor	90% in 5 days	India	[82]
*Pseudomonas* sp. But2	Butachlor	100% after 30 h	Vietnam	[83]
*Acinetobacter baumannii* DT	Propanil	100% after 48 h	Vietnam	[83]
*Achromobacter xylosoxidans*	2,4-D	95.38% after 96 h	Nigeria	[84]
*Pseudomonas* strain PD1	Pendimethalin	77.05% in 30 h	India	[85]
*Bosea* sp. strain P5 and *Alicycliphilus* sp. PH-34	Propanil	100% in 144 h	China	[86]
*Arthrobacter* sp. SVMIICT25, *Sphingomonas* sp. SVMIICT11 and *Stenotrophomonas* sp. SVMIICT13.	2,4-D	81–90% in 12 days	India	[66]
*Microbacterium sulfonylureivorans* sp. nov.	Nicosulfuron	69.56% within 7 days	China	[87]
*Stenotrophomonas rhizophila* CASB3	Diuron	94% in 42 days	Chile	[88]
*Bacillus pseudomycoides* D/T, *Bacillus simplex*/*Bacillus muralis* D/N	Diuron	54% and 51% in 46 days, respectively	Kenya	[89]
*Streptomyces heliomycini* C1	Metribuzin	73.06% after 15 days	Algeria	[90]
*Bacillus licheniformis* and *Bacillus megaterium*	Atrazine	98.60 and 99.60% after 7 days, respectively	China	[91]
*Pseudomonas putida*	Butachlor	100% in 15 days	India	[92]
*Acinetobacter* sp. GC-A6	Alachlor	100% in 48 h	South Korea	[93]
*Pseudomonas nicosulfuronedens* LAM1902	Nicosulfuron	99% after 6 days	China	[94]
*Bacillus pumilus* and *Bacillus subtilis*	Terbutylazine	95 and 98% within 6 days, respectively	China	[95]
*Pseudomonas stutzeri*	Atrazine	87% after 3 days	India	[96]
*Cupriavidus oxalaticus* strain X32	2,4-D	100% in 3 days	China	[67]
*Amycolatosis nivea* La24	Mesotrione	100% within 48 h	China	[97]
*Pseudomonas putida* Strain Ch2	Glyphosate	ND	Russia	[98]
*Cupriavidus campinensis*	2,4-D	94.69% after 6 days	Nigeria	[99]
*Bacillus* sp. Za	Fluoroglycofen	100% in 48 h	China	[100]
*Bacillus* sp. Za	Lactofen	100% in 36 h	China	[100]
*Dehalococcoides mccartyi* and *Desulfitobacterium hafniense*	2,4,5-trichlorophenoxyacetic acid (2,4,5-T)	ND	Germany	[101]
*Arthrobacter aurescens* CTFL7	Trifluralin	92% after 60 days	Spain	[102]
*Bacillus* sp. LY05	Butralin	85.43% after 30 days	China	[103]
*Bacillus subtilis* CZ1	Pendimethalin	58.85%	China	[104]
*Burkholderia* sp. F7G4PR33–4	Pendimethalin	65% in 15 days	Brazil	[105]
*Pseudomonas* strain PD1	Pendimethalin	77.05% in 1.25 day	India	[85]
**Fungi**				
*Clavispora lusitaniae* YC2	Pendimethalin	74% in 8 days	China	[106]
*Aspergillus* 2B112	Glyphosate	36.48 ± 0.01 in 14 days	Brazil	[107]
*Penicillium* 4A21	Glyphosate	42.72 ± 0.02 in 14 days	Brazil	[107]
*Penicillium 2A31*	Glyphosate	34.91 ± 0.02 in 14 days	Brazil	[107]

ND = Not determined.

**Table 2:** Mechanisms involved in herbicide degradation [40].

Degradation Mechanism	Outcome
1. Direct decomposition of herbicides through adaptation where herbicide compounds serve as energy sources (catabolism).	Repeated application of same herbicide results in faster degradation, such as atrazine [123]. Serious consequences may also arise such as persistence of some specific herbicides, e.g., atrazine and nicosulfuron residues in soil [124].
2. Accidental transformation through peripheral metabolic process (co-metabolism).	All herbicides may be degraded by this mechanism [125].
3. General activities by microorganisms such as modification of pH, production of different free radicals and other reactive compounds.	Degradation of herbicides due to the influence of microorganisms on biological and non-biological reactions [126].

**Table 3:** Effect of various factors (physical, chemical, structural, microbial and environmental) on the rate of degradation of herbicides. Updated from [138].

Factors	Properties	Degradation Rate	
		Rapid Degradation	Slow Degradation
Physical	Water solubility	Soluble	Insoluble
	Size	Relatively small	Relatively large
	Soil adsorption	Lower	Higher
	Soil moisture and temperature	Optimum	Both higher and lower values
	Origin	Biological	Synthetic or artificial
Structural	Functional group substitutions	Few	More
	Molecular weight and size	Low-molecular-weight and simple compounds	High-molecular-weight and complex compounds
	Substitutions on organic molecules	Alcohols, aldehydes, acids, esters, amides, amino acids	Alkanes, olefins, ethers, ketones, dicarboxylic acids, nitriles, amines, chloroalkanes
	Presence of polar functional groups	Presence	Absence
Chemical	Chemical reactivity	Presence of reactive groups (amines, esters)	Absence
	Volatility	low volatility	High volatility
Microbial	Microbial density and diversity	Higher microbial population	Lower microbial population
	Co-metabolism and nutrient availability	Sufficient nutrient available	Not available
Environmental	Oxygen availability	Aerobic conditions	Anaerobic conditions
	Soil amendments	Application of soil amendments (compost, biochar, etc.)	No application

**Table 4:** Recent advancements in microbial degradation of herbicides.

Name of Approach	Techniques	Objective	Outcome	References
Identification and Characterization	Culture methods, 16S rRNA sequencing, biodegradation trials	Isolation of novel bacterial strains and consortia capable of degrading specific herbicide	Identification of potential degraders (microorganisms)	[65]
Metagenomics and Next-Generation Sequencing (NGS)	Shotgun sequencing, Oxford Nanopore, PacBio, single-cell genomics	Analysis of the total microbial communities present in soil post herbicide application	Identification of non-culturable microorganisms, microbial diversity, and functional profiling	[210]
Biochemical and Enzymatic assays	Enzyme isolation, spectrophotometry, chromatography, mass spectrometry	Identification of enzymes responsible for degradation of herbicides	Understanding enzymatic pathways for microbial degradation	[211,212,213]
Stable Isotope Probing (SIP)	Isotopic labelling (13C, 15N), DNA/RNA extraction, sequencing	Detecting herbicide-derived isotopes in microbial DNA/RNA to identify potential degraders	Direct relation of microorganisms to degradation mechanism	[214,215]
Metabolomics and Mass Spectrometry	LC-MS, GC-MS, NMR spectroscopy	Characterization of herbicide metabolites to understand degradation pathways	Pathway analysis and identification of herbicide metabolites	[65,216]
Microcosm and Field Experiments	Herbicide residue analysis, microbial community profiling	Controlled laboratory or field experiments to monitor herbicide degradation and microbial response upon exposure	Microbial degradation processes under various environmental conditions	[146,217]
Bioinformatics and Systemic Biology	Genomic/transcriptomic data analysis, metabolic modelling	Integration and modelling of multi-omics data to understand degradation networks	Predictive insights for improving microbial degradation strategies	[29,218]
Transgenic microorganisms	Cloning and expression of the degradation gene into the engineered bacterium	Development of engineered bacteria for the degradation of highly persistent herbicides	Engineered bacteria degraded herbicides faster under optimized degradation conditions	[219,220]

## Data Availability

No new data were created or analyzed in this study. Data sharing is not applicable to this article.
